# Primary Cytomegalovirus Infection Causing Guillain-Barré Syndrome in a Living Renal Allograft Recipient

**DOI:** 10.1155/2017/7264793

**Published:** 2017-11-20

**Authors:** Massini Merzkani, Ezra Israel, Mala Sachdeva

**Affiliations:** Division of Kidney Diseases and Hypertension, North Shore University Hospital and Long Island Jewish Medical Center, Hofstra Northwell School of Medicine, Great Neck, NY, USA

## Abstract

Guillain-Barré Syndrome (GBS) is a common acute autoimmune polyneuropathy in adults. There have been few reported cases of Guillain-Barré Syndrome associated with active cytomegalovirus (CMV) infection in renal transplant recipients. Here we present a case of active CMV viremia inducing Guillain-Barré Syndrome in a renal transplant recipient. We discuss the treatment regimen utilized. Furthermore, we performed a review of the literature and discuss the cases of CMV induced GBS in renal transplant recipients.

## 1. Introduction

In solid organ transplantation, neurologic complications are not uncommon. Guillain-Barré Syndrome (GBS), also called acute inflammatory demyelinating polyneuropathy, is a common form of acute neuropathy in adults. Worldwide, its incidence is 1.3 per 100,000 population [1]. GBS carries a high incidence in bone marrow transplant recipients [2]. In kidney transplant recipients, GBS has been a rare occurrence and optimal treatment in this population has not yet been defined. On the contrary, CMV in kidney transplantation is not uncommon. Long-term CMV infection in a renal transplant recipient increases the risk for allograft failure and mortality [3]. The first case of Guillain-Barré Syndrome occurring in association with cytomegalovirus (CMV) infection was first reported in a renal transplant recipient in 1970 [4]. Second case was reported by Bale et al. 1980 of an active CMV infection inducing Guillain-Barré Syndrome [5]. There have been approximately a total of eleven cases that had this association worldwide in renal transplant recipients.

Optimal treatment for Guillain-Barré Syndrome in the immunosuppressed kidney transplant recipient is still unknown. Here we report a case of CMV induced GBS in a living unrelated renal transplant recipient who was treated with good clinical response with the combination of oral valganciclovir, plasma exchange, and intravenous immunoglobulins (IVIG).

## 2. Case

This is a 47-year-old Caucasian male with a history significant for a living unrelated renal transplant two years prior to presentation and recent acute cytomegalovirus (CMV) infection. He presented with a two-day history of worsening, ascending lower extremity weakness, numbness, and tingling. The weakness had worsened rapidly to the point where he had difficulty ambulating and imbalance. Seven days prior to presentation, he had first presented with fever, chills, myalgias, and headache of one-week duration. Work-up at that time revealed acute CMV infection and he was started on oral valganciclovir at treatment doses. On review of systems, the patient complained of mild shortness of breath that worsened on exertion. He denied fever, chills, nausea, vomiting, diarrhea, lightheadedness, cough, lower extremity edema, or any changes in his urine output. He denied any recent travel, sick contacts, recent history of insect bites, or vaccinations.

His past medical history was significant for hypertension, dyslipidemia, asthma, and a distant history of melanoma. The etiology of his end stage kidney disease was biopsy proven focal segmental glomerulosclerosis presumed to be secondary to anabolic steroid use. He had a living unrelated kidney transplant two years prior to presentation. Induction was with steroids and basiliximab. His posttransplant course was complicated by biopsy proven BK Virus Nephropathy one year after transplant for which he was successfully treated with reduction in immunosuppression and intravenous immunoglobulin. At the time of transplantation, the donor was CMV positive, and the recipient was CMV negative. He received prophylaxis with valganciclovir for CMV more than one year. His current immunosuppression regimen consisted of prednisone 5 mg daily, tacrolimus 1.5 mg every twelve hours, and mycophenolate mofetil 500 mg every twelve hours.

On physical examination, the patient appeared anxious but was awake, alert, and oriented to person, place, and time. Vital signs included a blood pressure of 137/98 mmHg, pulse rate of 81/min, temperature of 97 degrees Fahrenheit, respiratory rate of 16 breaths/min, and a pulse oximetry of one hundred percent on room air. Physical examination was significant for a neurological examination, which demonstrated decreased motor strength in proximal and distal muscles in both upper and lower limbs (grades 3/5 and 2/5, resp.). He had absent deep tendon reflexes in his lower extremities. His gait could not be assessed due to lower extremity weakness and inability to ambulate. Two days before, his neurological exam was noncontributory except for decreased temperature sensation in his hands and feet.

Laboratory data from one week prior to presentation included mild thrombocytopenia, mild increase in liver transaminases, and a stable creatinine. Diagnosis of acute CMV infection was made based on a positive serum CMV IgM titer of 149 au/ml (normal < 30 au/ml), negative CMV IgG, and CMV PCR with 4800 copies/ml. Lumbar puncture and other serologic work-ups were negative. MRI of the brain done revealed no acute changes. Due to a diagnosis of acute CMV infection, he was started on oral valganciclovir. Laboratory data at this presentation included white blood cell count 5.9 k/microliter; hemoglobin 16.8 grams/deciliter; platelets 312 k/microliter; sodium 139 mmol/liter; potassium 3.6 mmol/liter; chloride 105 mmol/liter; bicarbonate 23 mmol/liter; blood urea nitrogen 17 mg/dl; and creatinine 1.28 mg/dl (at baseline). Urine heavy metal screen was negative for arsenic, cadmium, mercury, or lead. HIV, HTLV I/II, HBV, HCV, HSV, EBV, and HHV-6 PCR analyses were all negative. Lumbar puncture showed cerebrospinal fluid protein at 63 mg/dL, glucose of 68 mg/dL, cell count of 1, and albumin of 52.7 mg/dL. CMV PCR was positive in the CSF. Serum CMV PCR was 4000 copies/mL.

Based on positive serum and cerebrospinal fluid CMV PCR, the typical albuminocytologic dissociation of CSF analysis, and progressive neurological manifestations, a diagnosis of CMV associated Guillain-Barré Syndrome was made.

The patient was on an immunosuppression regimen that consisted of prednisone 5 mg daily, tacrolimus 1.5 mg every twelve hours, and mycophenolate mofetil 500 mg every twelve hours. The mycophenolate mofetil was discontinued, and the Prograf dosage was reduced to target FK506 troughs of 5–7. In addition to reduction in immunosuppression, the patient received eleven treatments of plasma exchange over a course of two weeks (each exchange consisted of two plasma volumes each with albumin as the replacement) and a total of 1 g/kg body weight of intravenous immunoglobulin (IVIG) in two divided doses. He was continued on valganciclovir 900 mg orally two times a day. After 72 hours, the patient began to improve with increased motor strength and improved negative inspiratory flow and vital capacity measurements. Every successive plasma exchange treatment seemed to subjectively show a favorable response to his motor recovery and muscle strength. After a two-week hospitalization, the patient regained his ability to ambulate and he was subsequently discharged on oral valganciclovir. He was continued on treatment doses of valganciclovir at 900 mg orally two times a day as an outpatient. His serum CMV PCR was negative after two weeks of treatment. One month later, the patient returned with lower extremity weakness and was readmitted for a presumed relapse. At this time, his CMV PCR was still negative. The patient was then treated with a total of 7 plasma exchanges. He regained his motor strength once again after initiation of plasma exchange and subjectively reported progressive improvement with each treatment. The patient had no adverse reactions to the IVIG or to the plasmapheresis. He has been in remission after treatment. His renal function has remained stable through all of this.

## 3. Discussion

Guillain-Barré Syndrome has rarely been reported in the kidney transplant recipient. When clinical findings occur, it is usually preceded by either viral or bacterial infections that can be active during clinical presentation. Common pathogens that have been associated with GBS are* Campylobacter jejuni*, cytomegalovirus, Epstein-Barr virus, and* Mycoplasma pneumonia* [1]. Since CMV is common in the renal transplant recipient, it still remains unclear why GBS has not been reported more frequently in this population. Whether there is true underreporting of this association or whether there is some protective effect from the immunosuppression for GBS needs to be further elucidated. In the general population, morbidity and mortality with GBS are high with approximately 20% requiring artificial ventilation, 15–30% being left with severe disability and residual deficits, and about 5–10% requiring long-term use of a mechanical ventilator [13, 14]. Mortality can occur in 4–10% [15, 16]. In a transplant recipient, the course can be more severe; however, it is unpredictable. Thus, once clinically suspected, there should be prompt initiation of treatment to prevent further sequela.

The initial diagnosis of Guillain-Barré Syndrome is usually based upon the clinical presentation. The symptoms of GBS include ascending numbness, paresthesias, pain, or weakness in the limbs. GBS often can progress to ascending symmetric muscle weakness that can vary from mild difficulty with ambulation to nearly complete paralysis of all extremities, facial, and respiratory muscles [17]. Sensory involvement is common, but not as severe.

On physical examination, patients typically have motor weakness with absent or decreased deep tendon reflexes. Most patients usually have a lumbar puncture performed which can show increased cerebrospinal fluid (CSF) protein and usually have a normal CSF white blood cell count. Nerve conduction studies can confirm the diagnosis [17]. The presented patient showed typical clinical features and laboratory testing consistent with GBS. In addition, in his case there was a clear precedent acute CMV infection. See Table 1.

In the reported renal transplant recipients, most of the cases occurred in middle aged, males and the majority of the cases occurred within the first six months after transplantation, likely a reflection of net immunosuppression and opportunistic infection susceptibility at this time. Induction therapy was unknown for the majority, but most of the cases were on triple-drug regimen for their transplant which consisted of prednisone, a calcineurin inhibitor, and an antimetabolite.

Initial therapy in the nontransplant GBS patient is geared toward acute management of the ill patient. Usage of intravenous immunoglobulin (IVIG) or plasma exchange has shown to reduce time to recovery and still remain the most effective strategy to treat GBS [15, 17].

Treatment with intravenous immune globulin has been shown to be at least as effective as plasma exchange alone [15]. IVIG can be given at a dose of 0.4 g/kg bodyweight daily for five consecutive days [17]. Of the 11 cases reported in the literature in renal transplant recipients, six cases received IVIG. The plasma exchange regimen frequently consists of 5 plasma volume exchanges over a two-week period [17]. Interestingly, of the cases reported only one received plasma exchange. In solid organ transplantation for active CMV disease, it is common practice to decrease immunosuppression. This consists of reduction in prednisone, targeting a lower calcineurin inhibitor trough level, and decreasing the dosage of the antimetabolite. Addition of an antiviral medication is important to decrease active viral replication and decrease long-term outcomes.

Regarding general standard CMV prophylaxis in the renal transplant recipients, recipients are generally received prophylaxis for three to six months depending on the donor and recipient CMV status at the time of transplant. In one study, prophylaxis with valganciclovir 900 mg PO once a day for 200 days in kidney transplant recipients who are CMV mismatch donor positive and recipient negative showed a decrease in late CMV disease [18]. Regarding CMV acute infection, treatment depends on the severity of the disease and could include ganciclovir intravenously if the infection is severe or oral valganciclovir (900 mg twice a day) if the disease is mild [19]. Our patient received a total of one year of prophylaxis at the discretion of the physician due to his susceptibility to developing other opportunistic infections, such as BK Virus after transplant, and concern that he was being over immunosuppressed. However, to the authors surprise, the patient developed CMV even after receiving one year of prophylaxis when he did not seem to be over immunosuppressed.

In our literature review, the majority of the cases (seven out of the 11) reported with Guillain-Barré received treatment with intravenous ganciclovir for 14 to 21 days and there was improvement in most of the cases with recovery of neurological status. Our patient received oral treatment doses of antiviral therapy and did well. In patients who have recurrent CMV disease or fail to respond after adequate therapy genotypic testing should be a consideration. Other alternative therapies for CMV resistant strains could include foscarnet or cidofovir [19].

## 4. Conclusion

GBS presenting after CMV infection is not widely reported in a renal transplant recipient. Due to its debilitating nature, clinicians must be mindful of the presenting signs and symptoms as the diagnosis of GBS warrants rapid and timely initiation of therapy. Exact treatment is still unknown. In the cases thus reported, an antiviral medication (oral or intravenous) in combination with plasma exchange, IVIG, or both has been utilized. Our case is the first case with success using oral antiviral medications in combination with IVIg and plasma exchange. Because plasma exchange in patients with GBS has been shown to improve muscle strength early on and reduce the need for mechanical ventilation, we decided to treat our patient with this, which was atypical for other case reports [20]. Our patient showed daily improvement with every successive plasma exchange. In addition, after the short relapse, the patient responded very well to another short course of plasma exchange. In addition to decreasing the level of immunosuppression, we suggest using a combination of antiviral medication, plasma exchange, and IVIG in the treatment of CMV induced Guillain-Barre Syndrome in the renal transplant recipient.

## Figures and Tables

**Table 1:** Summary of cases with cytomegalovirus infection associated with Guillain-Barre Syndrome in renal transplant recipients.

Cases	Location	Age of recipient	Gender of recipient	Type of kidney transplant	CMV serostatus recipient/donor	CMV prophylaxis	Time after transplant when CMV infection developed	Immunosuppression regimen	Induction	Antiviral medication used for treatment	IVIG dose used for treatment	Use of plasma	Outcome
Drachman et al. 1970 [4]	US	28	Male	Cadaveric	NS/ NS	NS	5 months	Prednisone, azathioprine	None	No	No	No	Full recovery

Bale et al., 1980 [5]	USA	40	Male	Living	NS/ NS	NS	3 months	Prednisone and 3 days of radiation	NS	No	No	No	NS

Donaghy et al., 1989 [6]	UK	48	Male	Cadaveric	D+/R−	NS	7 weeks	Steroids, azathioprine, cyclosporine	NS	NS	NS	NS	NS

De Maar et al. 1999 [7]	Netherlands	58	Male	Cadaveric	D+/R−	NS	3 months	Cyclosporine, mycophenolate, prednisolone	None	Ganciclovir at a dose of 5 mg/kg every 12 h for 21 days	5 doses	No	Full recovery

El Sabrout et al. 2001 [8]	Texas	44	Male	Cadaveric	D+/R−	2-week preemptive course of ganciclovir followed by 3 months of oral acyclovir	2 years, 2 months	Mycophenolate mofetil, received for acute rejection of OKT3	NS	Steroids, no improvement	No	No	Partial recovery

El Sabrout et al. 2001 [8]	USA	47	Male	Cadaveric	D+/R−	NS	~7 years	Unknown, received for acute rejection of OKT3	NS	IV ganciclovir 5 mg/kg × 14 days	6 doses	No	Full recovery

Keithi-Reddy et al. 2007 [9]	India	48	Male	Cadaveric	D−/R−	NS	3 months post	Prednisolone, cyclosporine, mycophenolate mofetil	NS	IV ganciclovir 5 mg/kg every 12 hours for 21 days	5 doses	No	Full recovery

Alvarez et al. 2010 [10]	Spain	62	Male	Unknown	NS	NS	2.5 months	Steroids, tacrolimus, mycophenolate mofetil	NS	IV ganciclovir	5 doses	No	Full recovery

Alvarez et al. 2010 [10]	Spain	22	Female	Cadaveric	D+/R−	NS	5 weeks	Steroids, tacrolimus, mycophenolate mofetil	NS	IV ganciclovir	5 doses	4 cycles of plasmapheresis	Full recovery

Papasotiriou et al. 2013 [11]	Greece	28	NA	Cadaveric	D+/R−	IV ganciclovirfor 2 weeks, then 3 months with valganciclovir	190 days	Prednisone, tacrolimus, mycophenolate mofetil	Basiliximab, solumedrol	IV ganciclovir for 21 days. Then valacyclovir after 21 days. After 2 weeks, foscarnet	No IV IG	No	Had a relapse, then full recovery

Shaban et al. 2016 [12]	USA	62	Female	Cadaveric	D+/R−	Valganciclovir for 6 months	7 months	Tacrolimus and prednisone	Basiliximab and steroids	IV ganciclovir for 21 days	6 doses	No	Full recovery

NS = not specified, D = donor, and R = recipient.
